# How Early Can Pancreatic Tumors Be Detected Using NMR-Based Urine Metabolic Profiling? Identification of Early-Stage Biomarkers of Tumor Initiation and Progression in an Orthotopic Xenograft Mouse Model of Pancreatic Cancer

**DOI:** 10.3390/metabo15030142

**Published:** 2025-02-20

**Authors:** Tafadzwa Chihanga, Shenyuan Xu, Hannah N. Fultz, Jenna D. Nicholson, Mark D. Brombacher, Kayla Hawkins, Dan R. Fay, Maria M. Steil, Shuisong Ni, Michael A. Kennedy

**Affiliations:** Department of Chemistry and Biochemistry, Miami University, Oxford, OH 45056, USA; tchiha07@gmail.com (T.C.); xus2@zjut.edu.cn (S.X.); hannahnfultz@gmail.com (H.N.F.); jenna.nicholson16@gmail.com (J.D.N.); markdbrombacher@gmail.com (M.D.B.); kh891015@ohio.edu (K.H.); danroyfay@gmail.com (D.R.F.); nis@miamioh.edu (S.N.)

**Keywords:** pancreatic cancer, metabolomics, metabonomics, NMR, MiaPaCa-2, PDAC, mouse model, urine, orthotopic, xenograft

## Abstract

**Background:** Pancreatic cancer is the most lethal of all human cancers. The disease has no obvious symptoms in its early stages and in the majority of cases, the cancer goes undetected until it has advanced to the point that surgery is no longer a viable option or until it has metastasized to other organs. The absence of reliable and sensitive biomarkers for the early detection of pancreatic cancer contributes to the poor ability to detect the disease before it progresses to an untreatable stage. **Objectives:** Here, an orthotopic xenograft mouse model of pancreatic cancer was investigated to determine if urinary metabolic biomarkers could be identified and used to detect the early formation of pancreatic tumors. **Methods:** The orthotopic xenograft mouse model of pancreatic cancer was established by injecting human MiaPaCa-2 cells, derived from a male patient aged 65 years with pancreatic adenocarcinoma, into the pancreata of severe combined immunodeficient mice. Orthotopic pancreatic tumors, allowed to grow for eight weeks, were successfully established in the pancreata in 15 out of 20 mice. At the time of sacrifice, tumors were excised and histologically analyzed and the masses and volumes recorded. Urine samples were collected prior to injection, at one-week post injection, and every two weeks afterwards for eight weeks. **Results:** NMR-based metabolic profiling of the urine samples indicated that 31 metabolites changed significantly over the course of tumor initiation and growth. Longitudinal metabolic profiling analysis indicated an initial increase in activity of the metabolic pathways involved in energy production and/or cell synthesis by cancer cells as required to support tumor growth that was followed by a diminished difference between control and orthotopic mice associated with tumor senescence as the tumors reached 7–8 weeks post injection. **Conclusions:** The results indicate that NMR-based urinary metabolic profiling may be able to detect the earliest stages of pancreatic tumor initiation and growth, highlighting the potential for translation to human clinical studies.

## 1. Introduction

It is estimated that 66,440 people will be diagnosed with pancreatic cancer in 2024 and that 51,750 people will die from the disease in the US alone [1]. While only comprising ~3% of new cancer diagnoses, pancreatic cancer is the fourth leading cause of cancer-related deaths, making it one of the deadliest of all major cancers [1,2,3]. Only 13% of those who are diagnosed are predicted to still be alive after five years, and 72% of newly diagnosed people are predicted to die within the first year [1,4]. Unfortunately, the survival rates of those diagnosed with pancreatic cancer have only improved modestly over the last forty years [1,2,5]. Only 10% of cases are detected while the cancer is still confined to the pancreas and can be effectively treated by surgery [3]. The lack of a reliable early detection method is largely responsible for poor survival statistics after diagnosis.

Pancreatic ductal adenocarcinoma (PDAC) is the most common form of pancreatic cancer accounting for 95% of pancreatic cancer cases [6,7]. Pancreatic cancer is thought to emerge as a result of multiple genetic mutations. K-Ras mutations are present in 90% of pancreatic cancers, and these K-Ras mutations result in the activation of proliferative pathways that promote cancer progression [8]. Although K-Ras mutations are the most common in pancreatic cancer, up to 63 other genetic mutations were also identified [9]. PDAC is thought to progress from the formation of precancerous lesions known as PanINs [10,11] that originate from the metaplasia of pancreatic ductal epithelial cells [6] or by acinar to ductal metaplasia [12], and these PanINs eventually progress to PDAC. Pancreatic tumors characterized by a dense stroma, which is essential for tumor establishment and growth, and the tumor microenvironment is known to be hypoxic, requiring cancer cells to be adaptive for survival under these conditions [13]. Other adaptive mechanisms include the hijacking of nutrient sources from healthy cells to support tumor development [8,14,15].

### 1.1. Genetic Screening for PDAC

In recent years, researchers have made great strides in identifying genetic mutations that lead to pancreatic cancer [9]. In a genome wide associated study, Jones et al. identified core signaling pathways that possibly support pancreatic tumorigenesis using an array of genetic analysis techniques to analyze 24 pancreatic cancers [9]. By isolating and amplifying DNA from PDAC tumors, they were able to detect mutations, deletions, and amplifications that altered pathways that were an essential driving force for tumor growth [9]. In other studies, genetic mutations such as BRCA [16,17] and PALB2 [17,18,19] and conditions such as cystic fibrosis [20,21] and Peutz–Jeghers Syndrome [19] were shown to significantly increase the risk of developing pancreatic cancer. These genetic markers and conditions are used to screen for possible PDAC development, but the accuracy, cost, and accessibility remain a diagnostic deterrent.

### 1.2. Imaging Modalities for Detection of PDAC

Magnetic resonance imaging (MRI), colangiopancreatography, and endoscopic ultrasound (EUS) are other screening techniques utilized for PDAC diagnosis [19,22]. These techniques generally lack adequate sensitivity and specificity, resulting in low PDAC diagnostic yields [19,22]. Carbohydrate antigen 19-9 (CA19-9) is currently the most reliable clinical diagnostic biomarker for pancreatic cancer with approximately 85% specificity and 85% sensitivity [22,23,24]. The specificity and sensitivity of CA19-9 can be improved when coupled with other markers of pancreatic cancer [19,22].

### 1.3. Other Molecular Diagnostic Methods for PDAC Detection

Despite a growing effort by researchers to discover novel diagnostic approaches for the detection of pancreatic cancer using non-invasive biomarkers, reliable and robust clinical diagnostic biomarkers for the early detection of pancreatic cancer remain unavailable. Recently, some new diagnostic approaches for the detection of pancreatic cancer were suggested. For example, deregulated miRNAs were identified that potentially distinguish healthy individuals from those with pancreatic cancer [19,22,25]. Increased DNA methylation in pancreatic juice [26] and stool is also thought to be a potential new biomarker for the detection of pancreatic cancer [22]. A relatively new approach for the detection of cancer includes the use of liquid biopsy techniques to screen for circulating tumor cells and circulating tumor DNA [24], although circulating tumor cells are primarily responsible for metastatic development and thought to be rare in pancreatic cancer [22,27]. To date, these novel approaches also lack the sensitivity and specificity requirements for the clinical diagnosis of PDAC.

### 1.4. Proteomics-Based Detection of PDAC

Proteomics was also used to identify potential protein biomarkers of pancreatic cancer. Mannose-binding lectin 2 and myosin light chain kinase 2 were identified as possible serum biomarker of pancreatic cancer [28]. Other proteomic biomarkers include the regulatory subunit 8 of the 26S proteasome (PSMC5), the regulatory subunit 12A of protein phosphatase 1 (MYPT1), and the transferrin receptor (TFRC), which were significantly overexpressed in sera of PDAC patients compared to their healthy counterparts [28]. Multiple other potential protein biomarkers were identified using a variety of techniques including LC-MS, MALDI-TOF, Bio-Plex suspension array, and SDS-PAGE [27,29]. Validation of these biomarkers in larger studies is essential to increasing the number of clinically accepted biomarkers of PDAC.

### 1.5. Mass Spectrometry- and NMR-Based Metabolic Profiling-Based Detection of PDAC

Mass spectrometry (MS)- and nuclear magnetic resonance spectroscopy (NMR)-based sera metabolic profiling was explored as a means of identifying metabolites that can potentially be used for screening and early detection of pancreatic cancer. Ritchie et al. identified significant changes in sera long chain fatty acids and choline-related metabolites that distinguished pancreatic cancer individuals from healthy controls [30]. Their study also identified and validated PC-594, a long chain fatty acid, as a potential biomarker for identifying high risk individuals [30]. To increase specificity and sensitivity, multiple biomarkers can be used to distinguish control compared to pancreatic cancer samples [31]. Plasma glutamate, choline, 1,5-anhydro-d-glucitol, betaine, and methylguanidine were validated as diagnostic markers of pancreatic cancer, with 98% sensitivity and 83% specificity [31]. Though several potential metabolic biomarkers of pancreatic cancer were identified, validation of these metabolites is essential before they can have any clinical impact. A study by Mehta et al. assessing the predictive power of multiple previously identified biomarkers of pancreatic cancer showed reduced accuracy for discriminating pancreatic cancer from colorectal cancer samples [32].

### 1.6. Advantages and Limitations of NMR-Based Metabolic Profiling

NMR-based metabonomics has multiple advantages compared to some other technologies including reproducibility, ease in identification and quantification of metabolites [33], and minimal sample manipulation prior to data collection [34,35,36]. The main disadvantages of NMR-based metabolic profiling is its relatively poor sensitivity compared to mass spectrometry-based approaches and challenges in making complete assignments due to limitations in existing databases [37]. NMR serum metabonomics was used in the characterization of multiple human cancers including breast cancer, prostate cancer, and lung cancer [38,39,40]. Although many “omics” studies were used to search for biomarkers of pancreatic cancer, very few metabolic profiling studies have been reported [41,42]. One NMR-based metabolic profiling study of human urine samples distinguished the control and PDAC patients; however, the study included individuals of varying ages, gender, and cancer stages [42].

In the study reported here, an orthotopic mouse model was used to determine if, and at what stage of tumor development, urinary biomarkers could be used to detect the presence of a growing tumor in the pancreas. Specifically, human MiaPaCa-2 cancer cells were used to establish orthotopic xenograft pancreatic tumors in severely combined immunodeficient (SCID) mice. The SCID mice were considered a good selection for this study since they lack both T and B lymphocytes [43], and therefore allow for the formation of the complex tumor microenvironment over the course of tumor formation [43]. The MiaPaCa-2 cell line, established by Yunis et al., in 1977 [44], was derived from a PDAC from a 65 year old male and has been extensively characterized and validated as a cell line widely used in pancreatic cancer research, including its metastatic potential [45]. We previously studied the aberrant metabolism of MiaPaCa-2 cells in cell cultures [46,47], but those studies were limited by the fact that the metabolism of those MiaPaCa-2 cells did not reflect growth and metabolism in the context of a tumor environment nor did the metabolic profiling reflect the complex interaction of the tumor in the context of a living organism [46,48]. Transgenic mouse models of pancreatic cancer also exist and were studied by our group [10,11,49] and others [6,50,51,52]; however, such studies are also limited by the fact that the transgenic model usually relies on mutation of a single or few genes and therefore the transformation to cancer does not reflect the natural complexity of genetic alterations that accompany the broad natural diversity of tumors that occur in humans in which dozens of genes are often found to be mutated [9]. Furthermore, the metabolic profiles characterized in transgenic mouse models of cancer report on metabolites and metabolic changes that may be specific to murine metabolism, which may not accurately reflect human PDAC. Orthotopic xenograft mouse models of human cancers derived from human cancer cell lines are valuable because they allow researchers to study the characteristics of tumors composed of human cancer cells in the context of a living animal while still being able to control experimental variables [53,54]. The results reported here demonstrated that it was possible to detect significant changes in the profiles of urinary metabolites as the orthotopic tumors were established and grew. Metabolites elevated in the urine of mice in the early stages of tumor growth indicated increased energy metabolism and increased activity of metabolic pathways required to support cell proliferation and tumor growth.

## 2. Materials and Methods

### 2.1. Institutional Approval of Mouse Studies

All procedures involving mice were approved by both the ethics committee and the Institutional Animal Care and Use Committee at Miami University (Animal Welfare Assurance Number: D16-00100). The approved protocols were assigned IACUC Project Numbers: 889 and 893. All procedures were conducted in such a way as to minimize any suffering or discomfort experienced by the mice. Daily health monitoring of the mice was conducted by the Miami University’s Animal Resources and Care Facility. Researchers were notified if any immediate action needed to be taken to care for the animals. For orthotopic surgeries, the mice were anesthetized using isoflurane so that the mice were unconscious and unable to experience pain during the surgery. To initiate anesthesia, the mice were placed in a drop box containing 3–5% atmospheric isoflurane. Once anesthetized, the mice were placed on the surgical table and the surgery conducted while a nose cone administered 1–3% isoflurane to maintain anesthesia. After surgery, 0.05–0.10 mg/kg buprenorphine was injected subcutaneously between the mouse’s shoulder blades following suturing of the incision as a pain reliever. The mice were then allowed to recover for 15 min after surgery in a cage atop a warming blanket. An additional dose of 0.05–0.10 mg/kg buprenorphine was injected subcutaneously 12 h post-operation to minimize pain and suffering of the mice during the study.

### 2.2. Orthotopic Xenograft Mouse Model of Pancreatic Cancer

Twenty NOD.CB17-Prkdcscid/J SCID mice were purchased from Taconic (Hudson, NY, USA) and maintained in a barrier facility of Miami University according to institutional guidelines. MiaPaCa-2 were purchased from the American Type Culture Collection (Manassas, VA, USA). The study size was based on a statistical power calculation ensuring that statistically significant changes in NMR peak intensities could be detected for the weakest effect size category according to Goodpaster et al. [55]. MiaPaCa-2 cells were grown on the recommended medium, i.e., high glucose Dulbecco’s Modified Eagle Medium, (DMEM) supplemented with 10% fetal bovine serum (FBS) and 1% penicillin-streptomycin (ThermoFischer, Pittsburgh, PA, USA). MiaPaCa-2 cells were scraped from the culture flasks and the suspended cells were drawn into insulin needles. Each mouse was administered 5% isofluorane in a sealed box to induce anesthesia. Once the mouse was unconscious, it was placed on the surgical table with a nose cone administering isofluorane at a concentration of 1–3% to maintain anesthesia during surgery. The mouse’s left side from a dorsal view was shaved and the spleen was located and used to find the pancreas. The site of the incision was scrubbed with butadiene and then rinsed with 70% alcohol three times. A 1 cm incision was made through both cutaneous layers and the pancreas was exposed. A 10 µL volume of MiaPaCa-2 cells suspended in DMEM high glucose media was injected into the pancreas. For the control mice, sham surgery was performed and a 10 µL volume of DMEM high glucose media was injected into the pancreas. A cotton swab was used to apply pressure in order to minimize leakage of cells into the abdominal cavity. The pancreas was repositioned in the abdominal cavity and the incision was sutured closed. A 0.10 mg/kg dose of buprenorphine was injected subcutaneously after the incision was closed to mitigate pain. Mice were fed ad libitum, and were monitored daily for signs of distress. After eight weeks of urine collection, as described below, the mice were anesthetized and euthanized by a terminal blood draw collected by cardiac puncture according to approved procedures. By 8 weeks, 15 of the 20 mice had developed tumors and were included in the metabolomics analysis described below. The 5 mice that failed to develop tumors were excluded from the study.

### 2.3. Sample Collection and Processing

Following sham surgery or MiaPaCa-2 injection into the pancreata, urine samples were collected 1 week after surgery and every 2 weeks afterwards up until 8 weeks. Prior to sacrifice, blood was obtained via cardiac puncture and placed in a microtainer tube with K_2_EDTA (ThermoFischer, Pittsburgh, PA, USA). The blood was spun down in a centrifuge at ≥ 22,000× *g* and 4 °C for 20 min. The plasma was removed and stored in the −80 °C freezer for further NMR data analysis. The mice were sacrificed and the pancreata were harvested and stored. Samples of fixed pancreas tissue were embedded in paraffin for subsequent histological analyses.

### 2.4. Histology

Sagittal sections (5 μm) of the pancreata were examined to confirm tumor burden using standard hematoxylin and eosin (H&E) staining. HMGA expression was determined as previously described by Veite-Schmahl et al. [9]. All micrographs were taken using an Olympus AX70 light microscope with a Cool Snap K4 digital Camera (Photometrics, Tucson, AZ, USA).

### 2.5. NMR Data Collection

The urine samples were stored at −80 °C after collection. The samples were thawed on ice, then prepared for NMR analysis. A 1 mL aliquot of each sample was pH corrected to 7.4, and then centrifuged and buffered as previously published by Romick-Rosendale et al. [56,57]. D_2_O was added to the urine samples to reach a final concentration of 10%, trimethylsilylpropanoic acid (TSP) was added to a final concentration of 10 mM as a chemical shift reference standard, and the samples were spiked with EDTA to prevent trace paramagnetic metal broadening of proton NMR resonances. All NMR spectra were recorded on a Bruker Avance TM III spectrometer operating at 600 MHz. All experiments were conducted at 298 K using 5 mm NMR tubes (Norell, Morganton, NC, USA). A standard ^1^H 1D presaturation (zgpr) experiment was collected to assess the sample shimming. Samples were considered to be adequately shimmed when the TSP peak linewidth was < 1 Hz. Once the sample was adequately shimmed, the 1D first increment of a NOESY (noesypr1D) was collected to determine if there were changes in lipid composition and the CPMG (cpmgpr1d) experiments were recorded to allow quantitation of metabolite changes. All spectra were processed as previously reported by Romick-Rosendale et al. [57]. All spectra were referenced in the ^1^H dimension relative to an internal TSP standard set to 0.0 ppm. Two-dimensional ^1^H-^1^H TOCSY and ^1^H-^13^C HSQC spectra were collected as previously described by Chihanga et al. [58]. In brief, the 2D ^1^H-^1^H TOCSY and ^1^H-^13^C HSQC spectra were recorded at a constant temperature of 298 K using HSQC (hsqcetgpsi). The 2D ^1^H-^1^H TOCSY was collected using the TOCSY (mlevgpph19) pulse sequence at 298 K. The data were processed with Topspin 3.2 (Bruker BioSpin, Billerica, MA, USA).

### 2.6. Multivariate Statistical Analysis and Statistical Significance of Individual Buckets

The Bruker AMIX software v.3.9 (Analysis of MIXtures software, Bruker Biospin, Billerica, MA, USA) manual bucketing tool was used to bucket spectral resonances. Buckets in each spectrum were normalized to total intensity prior to statistical analysis. It should be noted, however, that despite the fact that equal urine volumes were used for each sample, normalization to total intensity can increase the variance in control and study peak intensities [37]; however, all of the unnormalized and normalized binned data were deposited in a public database (https://figshare.com, accessed on 17 January 2025) to enable other researchers to reanalyze the data if desired (https://figshare.com/articles/dataset/Raw_NMR_metabolic_profiling_data/28230212, accessed on 17 January 2025). Bonferroni corrected alpha values (equal to 0.05 divided by the number of buckets) were calculated for each comparison to ensure maintenance of a 5% false positive rate when performing multiple comparisons, i.e., *p*-value tests, to a single dataset, which in this case refers multiple *p*-value tests applied to a single set of control and study NMR spectra. The details regarding the execution of the Bonferroni correction are described in detail in Goodpaster et al. [55]. PLS-DA was performed using the SIMCA-P ver. 11.0 software package (Umetrics, Umea, Sweden). Metabolite concentrations were measured relative to the TSP internal standard concentration. Calculation of the Mahalanobis, F-value and F-critical values, as previously described by Goodpaster et al. [59] enabled a quantitative analysis of whether or not the separation between the two distributions of points in PLS-DA score plot, one distribution for the control mice NMR spectra and one distribution for the study mice NMR spectra, was statistically significant or not. In the PLS-DA score plot, each point represents the spectral content of an entire NMR spectrum. As with the *p*-value tests, the null hypothesis is that the control and study spectra belong to the same group. When the F-value is greater than the critical F-value, one can reject the null hypothesis and conclude that the groups of points in the PLS-DA score plot belong to two distinct groups, based on a rigorous statistical test. Without application of the Mahalanobis calculation and the F-value calculations, one is unable to make any quantitative determination about the relative distribution of the two groups of scores in the PLS-DA score plot. The critical F-values were calculated using the online server, https://www.danielsoper.com/statcalc/calculator.aspx?id=4, accessed on 17 January 2025, and the input for the degrees of freedom were determined as follows: degrees of freedom 1 was equal to the number of the samples in the control group—1; and the degrees of freedom 2 was equal to the number of samples in the study group—1. The instructions for how to calculate the degrees of freedom can be found at the following link: https://stattrek.com/probability-distributions/f-distribution, accessed on 17 January 2025.

### 2.7. Identification and Quantification of Metabolites

Metabolite identification and quantification were assessed as previously described by Chihanga et al. [58] and the confidence in the metabolite assignments was evaluated using RANCM scores [33]. Briefly, initial metabolite assignments were made using Chenomx v.8.1 (Chenomx Inc., Edmonton, AB, Canada) and confirmed using the Complex Mixture Analysis by NMR (COLMAR) software [60] that involved matching experimental spectra to reference databases of metabolites. Two-dimensional NMR spectra (^1^H-^1^H TOCSY and ^1^H-^13^C HSQC) were also used to confirm assignments and to define the RANCM scores [33].

### 2.8. Metabolic Pathway Analysis

The pathway analysis module of MetaboAnalyst (http://www.metaboanalyst.ca/faces/home.xhtml, accessed on 17 January 2025) was used to identify metabolic pathways that involved metabolites that experienced statistically significant changes in concentration over the course of tumor growth.

## 3. Results and Discussion

### 3.1. Histological Examination of Pancreatic Tumors

**Figure 1:**
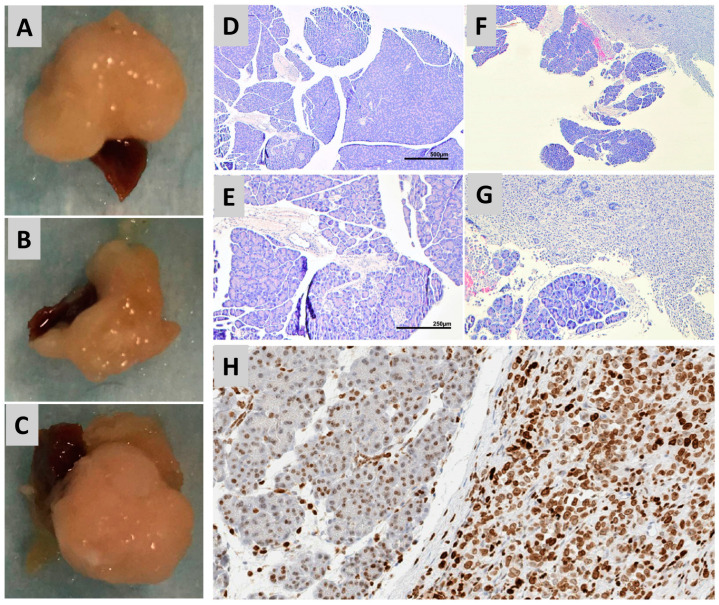
Morphological and histological analysis of pancreatic tissue in control mice and mice with PDAC pancreatic tissue sections. (**A**–**C**) Representative examples of orthotopic pancreatic tumors excised from mice injected with MiaPaCa-2 cells. Dark magenta colored structure is spleen attached to pancreas. Relatively large pale light colored masses represent tumor tissues. (**D**) Low magnification control showing healthy pancreatic tissue. (**E**) Low magnification images showing both healthy tissue and invasive PDAC. (**F**) High magnification images of healthy pancreatic tissues highlighting normal acinar islet and ductal health structures. (**G**) High magnification juxtaposition of healthy structured acinar cells and tumor cell invasion of healthy tissue. (**H**) Immunohistochemistry of orthotopic tumor surrounded by normal acinar tissue staining for HMGA-1. Dark brown staining in nuclei of tumor cells indicates strongly elevated expression of HMGA-1 in cancer cells in comparison to relatively weak staining in nuclei of acinar cells.

Injection of human MiaPaCa-2 cells into the pancreata of the mice was successful in producing palpable local tumor growth (Figure 1) in 75% of the SCID mice (15 out of 20 mice) based on assessment at eight weeks after surgery. Tumors were typically palpable by six weeks post injection of MiaPaCa-2 cells into the pancreata. The sizes of these tumors ranged from 0.05 to 1.40 cm^3^ with a mean tumor volume of 0.48 cm^3^. The weight of these tumors ranged from 0.38 to 1.64 g with an average tumor weight of 0.89 g. After euthanasia, the tumors (if present) were easily identifiable during dissection based on their morphology and consistency, as illustrated in Figure 1A–C. Normal pancreas tissue has a flexible and spongy consistency as opposed to the firm and rigid consistency of a tumor, which develops due to the desmoplastic stromal reaction that leads to the formation of dense fibrous tissue.

The tumors obtained during dissection were verified as pancreatic adenocarcinoma using histological analysis. Healthy pancreas tissue is composed of acinar cells with basally located nuclei giving the appearance of well-differentiated tissue (Figure 1D,E). This is starkly contrasted with neoplastic tissue, which is composed of unorganized nuclei that are no longer basally located, indicating poor differentiation (Figure 1F,G). Tumor invasion into the healthy acinar tissue can be easily identified based on the strong contrast in tissue and cell morphology. Poorly differentiated neoplastic tissue was identified in all of the mice that had visible evidence of pancreatic tumors. Tumor tissues were also screened for HMGA1 expression. The nuclei of normal healthy acinar tissue exhibited relatively weak staining for HMGA1 compared to the nuclei of the cancer cells comprising the tumors, which stained strongly for HMGA (Figure 1H). These results are consistent with our previous reports that indicate that MiaPaCa-2 cells in cell culture also exhibit strong expression levels of HMGA1 [46,61] and with our previous observations that HMGA1 levels are elevated in the nuclei of precancerous pancreatic intraepithelial neoplasia cells in the Ptf1a-Cre; LSL-KrasG12D transgenic mouse model of pancreatic cancer [10].

### 3.2. ^1^H NMR Spectroscopy-Based Metabolic Profiling Analysis of Tumor Progression

**Figure 2:**
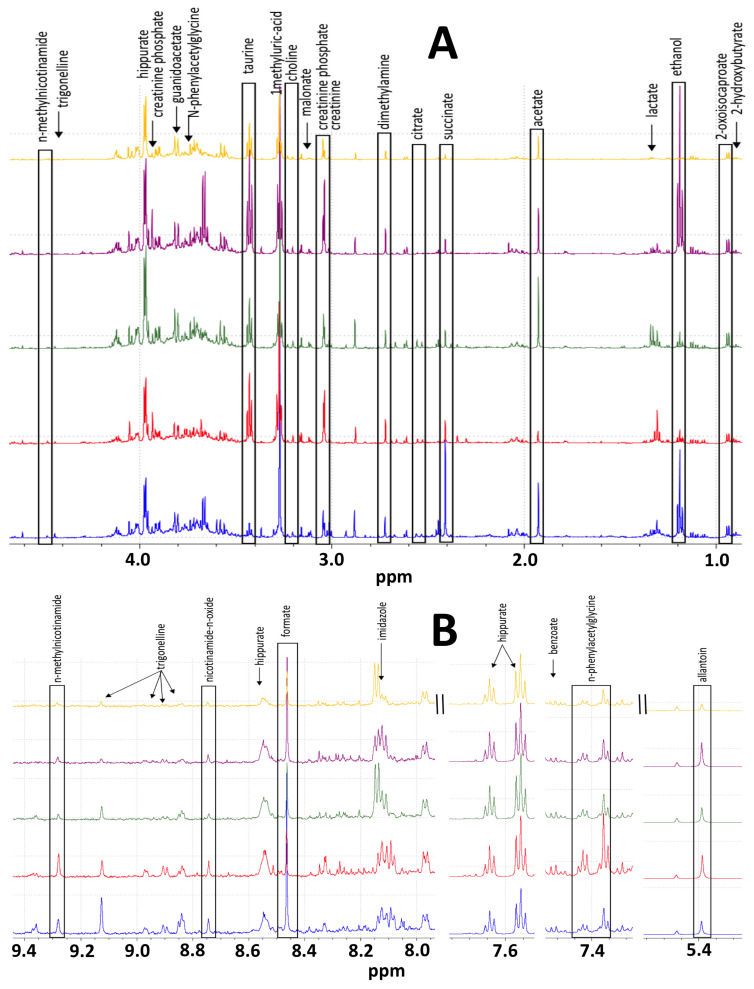
Differences in urine NMR spectra illustrating changes in metabolite concentrations as result of tumor progression. Representative one-dimensional ^1^H NMR spectra shown in (**A**) aliphatic regions and (**B**) aromatic region of urines collected from control sham mice (at top) and collected 1 week, 3 weeks, 5 weeks, and 7 weeks post orthotopic injection of cancer cells descending from control spectrum shown at top, respectively.

**Figure 3:**
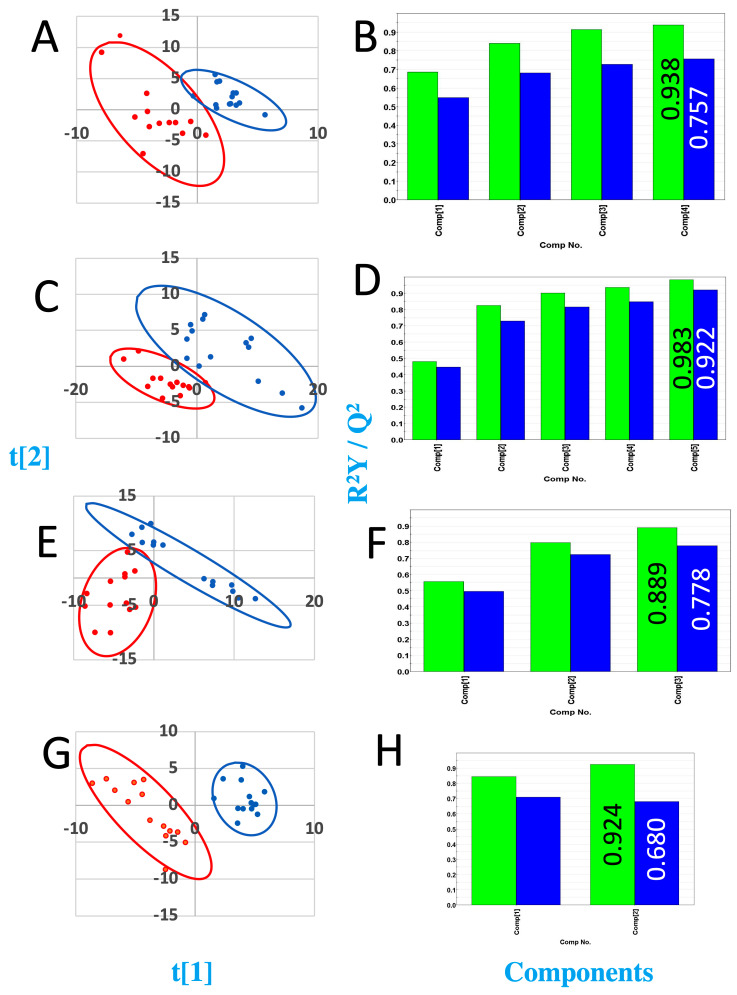
PLS-DA scores and R^2^/Q^2^ plots for urine samples from orthotopic, xenograft mice in comparison to sham surgery mice. (**A**,**C**,**E**,**G**) PLS-DA score plot for comparison of urines from sham mice and orthotopic cancer cell line injected. Blue points represent “control” samples and red points represent “study” samples. The blue and red ovals indicate the 95% confidence intervals for the distributions. (**B**,**D**,**F**,**H**) Graphs of cumulative R^2^Y values (green bars), and Q^2^ values (blue bars), obtained from cross-validation of data using repeated calculations withholding 1/7 of data.

NMR data were collected for urine obtained from the control mice that had a sham surgery with cell culture media injected into their pancreata and for urine collected from the orthotopic study mice that had MiaPaCa-2 cancer cells suspended in cell culture media injected directly into their pancreata. Urine samples were collected from the sham control mice and from orthotopic study mice at 1 week, 3 weeks, 5 weeks, and 7 weeks post-surgery. The mice were typically housed in metabolism cages at least two times separated by 2–3 days during the week of data collection, and the mice were euthanized at 8 weeks after surgery. Representative spectra of urine samples from each time point following surgery are shown in Figure 2. One-dimensional ^1^H NMR spectra were analyzed using multivariate statistical methods to assess group clustering and using statistical significance testing based on *p*-value calculations to identify resonances for metabolites whose concentrations changed significantly between the sham and orthotopic urine samples. PLS-DA VIP scores and *p*-values were used to identify metabolites that distinguished the control and orthotopic model mice, and that represented potential biomarkers of tumor initiation and progression. PLS-DA of the NMR spectra of the urine samples performed for each time point between the sham and study mice are presented in Figure 3.

### 3.3. Week 1 Analysis

The PLS-DA score plot for the week-1 orthotopic to sham surgery control group comparison is shown in Figure 3A. The Mahalanobis distance for cluster separation was 4.46 and the F-value was calculated to be 91.72, which when compared to the critical F-value = 2.217 (degrees of freedom 1 = *n* − 1 = 18 and degrees of freedom 2 = *n* − 1 = 18), indicated that the separation between the NMR spectra of the sham surgery and orthotopic mice urine samples was statistically significant after 1 week of tumor growth. The *p*-value calculations indicated that 17 of 114 resonances experienced intensity changes that were statistically significant. Cross-validation indicated good model quality (R^2^Y (cum) = 0.938) and strong predictive power based on a cumulative Q^2^ value of 0.757 (Figure 3B).

The significant changes in resonance intensities corresponded to changes in urine concentrations of 27 potential metabolites including 1-isovaleric acid, 2-hydroxybutyrate, ethanol, dihydrothymine, lactate, alanine, acetate, methionine sulfoxide, succinate, methylamine, 2-oxoisocaproate, dimethylamine, creatine phosphate, creatinine, phosphoryl choline, taurine, trimethylamine N-oxide, 1-methyluric acid, dimethylglycine, 6-anhydro-beta-d-glucose, allantoin, NADP, cis-aconitate, n-phenylacetyl glycine, Hippurate, trigonelline, and n-methylnicotinamide (Supplementary Materials Table S1). Area under the curve (AUC) analysis, which indicates accuracy of prediction to group belonging, indicated a close agreement with the significant metabolite increases based on the *p*-value analysis, as expected [37].

**Figure 4:**
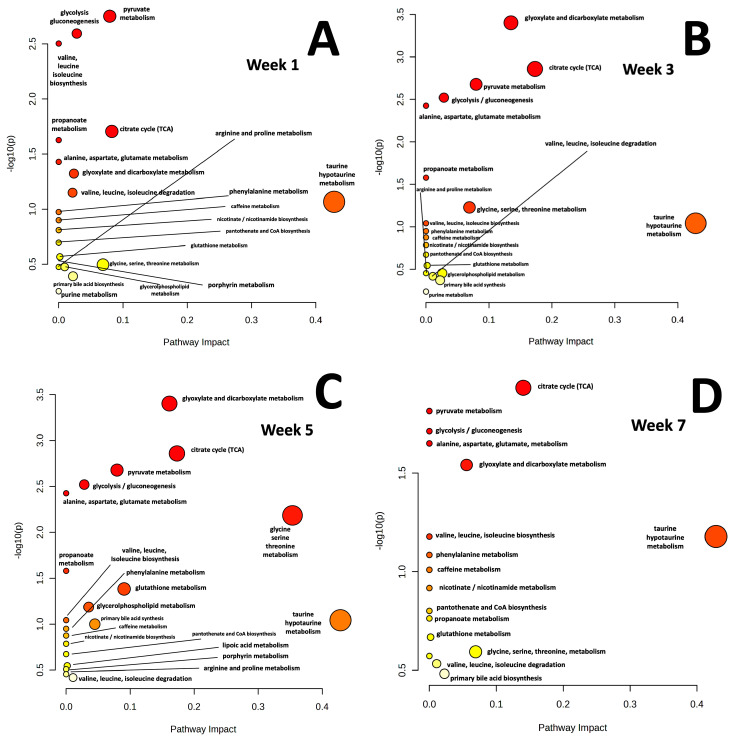
Metabolic pathways map. (**A**) Week 1 pathway analysis. (**B**) Week 3 pathway analysis. (**C**) Week 5 pathway analysis. (**D**) Week 7 pathway analysis. Pathway nodes are colored based on *p*-value determined by impact of pathway, determined by number of metabolites identified in pathway, with darker colors and larger circles indicating more metabolites. Compound lists used to generate pathway analyses are included in Supplementary Table S2.

A pathway analysis of the 27 metabolites whose concentrations changed in the urine after the first week following orthotopic injection of MiaPaCa-2 cells into mice pancreata indicated that the three most significantly affected pathways included pyruvate metabolism, glycolysis/gluconeogenesis, and valine, leucine, isoleucine biosynthesis (Figure 4A). Elevated pyruvate metabolism, based on elevated urinary lactate levels, was consistent with the elevation of glycolysis since pyruvate is the end product of glycolysis. Increased glycolysis activity, also identified primarily based on elevated urinary lactate levels, is not surprising since cancer cells rely primarily on glycolysis for energy production, even in the presence of oxygen. Elevated gluconeogenesis, also associated with elevated urinary lactate levels, would be consistent with the breakdown of existing lipids and proteins to generate a source of glucose during the high energy needs of tumorigenesis. Elevation of these pathways is consistent with increased energy metabolism demands associated with rapid cancer cell proliferation and tumor establishment in the first week following orthotopic injection of the MiaPaCa-2 cells. In addition to the metabolites already mentioned associated with the three highest impact pathways, alanine, 2-hydroxybutyrate, 1-isovaleric acid, and n-methylnicotinamide were significantly elevated in week 1 urines. PDAC cancer cells were shown to require elevated levels of alanine as it supports PDAC growth and metabolism, possibly explaining its increased urine levels [62]. 2-hydroxybutyrate was shown to be elevated in individuals with pancreatic cancer, and was shown to be involved in a novel post-translational modification of lysine residues that may promote PDAC progression [63]. N-methylnicotinamide, a known metabolite required for tumorigenesis, may be elevated in the urine because it is produced by tumor cells and can inhibit T-cell function that can reduce T-cell killing function [64].

### 3.4. Week 3 Analysis

The PLS-DA score plot for the week-3 orthotopic to sham surgery control group comparison is shown in Figure 3C. The Mahalanobis distance for cluster separation was 4.46 and with a corresponding F-value of 59.19, which when compared to the critical F-value = 2.577 (degrees of freedom 1 = *n* − 1 = 13 and degrees of freedom 2 = *n* − 1 = 13), indicated that the separation between the sham surgery and orthotopic mice urine samples was statistically significant after 3 weeks of tumor growth. Cross-validation indicated good model quality (R^2^Y (cum) = 0.983) and strong predictive power based on a cumulative Q^2^ value of 0.922 (Figure 3D).

The analysis of week-3 orthotopic group urines indicated 46 experienced intensity changes that were statistically significant by *p*-value, with another 26 resonances significant by either AUC or VIP. These resonances corresponded to significant changes in urine concentrations of 29 potential metabolites compared to in the control urines, including 1-isovaleric acid, 2-hydoxybutyrate, ethanol, dihydrothymine, lactate, alanine, acetate, methionine sulfoxide, citrate, succinate, trimethylamine, dimethylamine, creatinine phosphate, creatinine, choline, taurine, trimethyl-N-oxide, 1-methyluric acid, glycolate, 6-anhydro-beta-d-glucose, allantoin, NADP, cis-aconitate, n-phenylacetylglycine, hippurate, trigonelline, n-methylnicotinamide, dimethylglycine, and imidazole (Supplementary Materials Table S1).

At three weeks post injection of MiaPaCa-2 cells, there were five metabolic pathways most significantly affected, including glyoxylate and dicarboxylate metabolism, the TCA cycle, pyruvate metabolism, glycolysis and alanine, aspartate, and glutamate metabolism (Figure 4B). The high significance of the glyoxylate and dicarboxylate metabolism pathway was supported by significant increases in urinary cis-aconitate, citrate, and acetate concentrations. The glyoxylate pathway converts glyoxylate, a product of breakdown of carbohydrates into metabolites eventually used to produce sugars like glucose. The dicarboxylate pathway uses compounds with two carboxylate groups like succinate for energy production and biosynthesis. The significance of the TCA cycle was supported by elevated levels of cis-aconitate, citrate, and succinate. The significance of pyruvate metabolism was supported by significantly elevated lactate. The significance of the glycolysis/gluconeogenesis pathway was supported by significantly elevated lactate levels. The significance of the alanine, aspartate, and glutamate metabolism pathway was supported by significant increases in alanine, citrate, and succinate concentrations in week 3 (Supplementary Materials Table S1). Increased activity of this latter pathway is associated with amino acid synthesis and biosynthesis in support of tumorigenesis. All five of these most significantly impacted pathways are implicated in the elevated energy production required for supporting tumor initiation and growth. In addition to the five significantly impacted pathways identified above, significant increases in NADP occurred, which is an essential molecule to support the biosynthesis of nucleic acids, proteins, and lipids in cancer cells, potentially explaining its elevated levels observed in week 3 [65].

### 3.5. Week 5 Analysis

The PLS-DA score plot for the week-5 orthotopic to sham surgery control group comparison is shown in Figure 3E. The Mahalanobis distance for cluster separation was 3.863 with a F-value of 64.964, which when compared to the critical F-value = 2.577 (degrees of freedom 1 = *n* − 1 = 18 and degrees of freedom 2 = *n* − 1 = 18), indicated that the separation between the sham surgery and orthotopic mice urine samples was statistically significant after 5 weeks of tumor growth. Cross-validation indicated good model quality (R^2^Y (cum) = 0.889) and strong predictive power based on a cumulative Q^2^ value of 0.778 (Figure 3F).

The analysis of week-5 orthotopic group urines indicated 34 experienced intensity changes that were statistically significant by *p*-value, with another 19 resonances significant by either AUC or VIP. These resonances corresponded to significant changes in urine concentrations of 31 potential metabolites compared to the in control urines, including lactate, acetate, 1-isovaleric acid, alanine, choline, trimethylamine, creatinine phosphate, methionine sulfoxide, taurine, trimethylamine N-oxide, 1-methyluric acid, benzoate, creatinine, dimethylamine, trigonelline, imidazole, and n-phenylacetylglycine, and a decrease in ethanol and dimethylglycine were observed in the study mice urines compared to control mice urines (Supplementary Materials Table S1).

Week-5 orthotopic mice continued to exhibit elevated urine concentrations of metabolites involved in energy metabolism in support of tumorigenesis, i.e., glyoxylate and dicarboxylate metabolism, the TCA cycle, pyruvate metabolism, glycolysis or gluconeogenesis, alanine, aspartate, and glutamate metabolism (Figure 4C), as discussed above.

### 3.6. Week 7 Analysis

The PLS-DA score plot for the week-7 orthotopic to sham surgery control group comparison is shown in Figure 3G. The Mahalanobis distance for cluster separation was 6.735 and the F-value was calculated to be 152.652, which when compared to the critical F-value = 2.577 (degrees of freedom 1 = *n* − 1 = 13 and degrees of freedom 2 = *n* − 1 = 13), indicated that the separation between the sham surgery and orthotopic mice urine samples was statistically significant after 7 weeks of tumor growth. Cross-validation indicated good model quality (R^2^Y (cum) = 0.924) and strong predictive power based on a cumulative Q^2^ value of 0.680 (Figure 3F).

The analysis of week-7 orthotopic group urines indicated 23 experienced intensity changes that were statistically significant by *p*-value, with another 23 resonances significant by either AUC or VIP. These resonances corresponded to significant increases in 23 potential metabolites compared to the in control urines, including 1-isovaleric acid, 2-hydroxybutyrate, ethanol, dihydrothymine, lactate, alanine, methionine sulfoxide, citrate, taurine, trimethyl-N-oxide, 1-methyluric acid, 6-anhydro-beta-d-glucose, NADP, cis-aconitate, n-phenylactelyglycine, hippurate, trigonelline, n-methylnicotinamide, dimethylglycine, imidazole, benzoate, creatine phosphate, and creatinine (Supplementary Materials Table S1).

By week 7, the physiology of the aging tumors, which were well established by this time and visible prior to excision, appeared to change from a phase of rapid growth following initiation to a state of senescence, reversing the trend of significantly elevated urinary metabolites involved in energy production and metabolic pathway intermediates known to support cell proliferation, indicated by a return to a metabolic pathway resembling that of the healthy control mice. While the same five energy-related pathways were identified as having high impact in the pathway analysis (Figure 4D), the relative increase in the metabolites was less than at 3 and 5 weeks, as discussed below. One noteworthy observation in the mice at week-7 was that the mice exhibited visual signs of weight loss consistent with diabetic wasting. One explanation for diabetic wasting is increased insulin resistance within the skeletal muscle tissue, which was previously identified in human PDAC patients [66]. Compromised pancreas function due to tumor growth would also be expected to disrupt the structural and functional integrity of the islets [67], thus impacting insulin production and digestive enzyme secretion, resulting in a pseudo diabetic state accompanied by physical wasting. This increased demand for energy metabolites would be expected to be associated with increased metabolic demands in the host, resulting in a concomitant decrease in excreted urinary metabolites, which was observed in the week-7 urine in comparison to the urinary metabolic profiles measured in the earlier week-3 and week-5 samples.

### 3.7. Longitudinal Analysis of Urine Metabolite Changes During Tumor Progression

**Figure 5:**
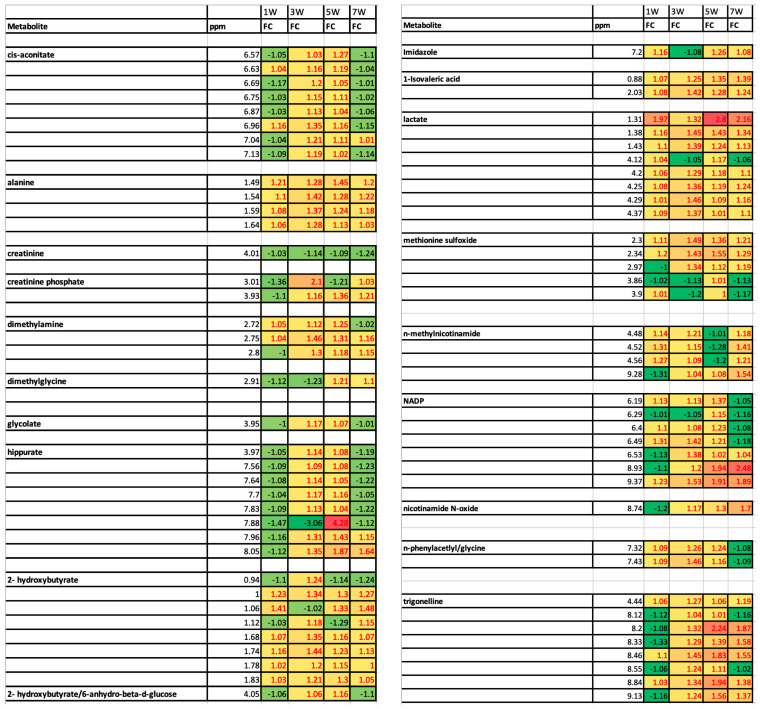
Heat maps of metabolic changes as function of tumor growth following orthotopic injection of human MiaPaCa-2 cancer cells into mice pancreata. Yellow to red coloring indicates increases in metabolite concentrations in urine of orthotopic, xenograft mice in comparison to that of sham surgery control mice. Light to dark green coloring indicates higher metabolite concentrations in urine of sham surgery control mice in comparison to those of orthotopic, xenograft mice.

Examination of Supplementary Materials Table S1 enables an analysis of trends in changes in urinary metabolite concentrations over the course of tumor development. A heat map analysis of a subset of metabolites that showed statistically significant changes is shown in Figure 5. Several of these metabolites exhibited a trend that their urinary levels were elevated reaching peak values in weeks 3 and 5 of tumor progression followed by a return toward the control mice urinary levels in week 7. Many of the metabolites that exhibited these trends are implicated in metabolic pathways involved in energy production and other pathways activated to support tumor growth, as discussed above. An analysis of the metabolic pathways active under nutrient-replete and nutrient-deprived conditions such as tumor environment as well as the tumor survival mechanisms triggered under nutrient-deprived conditions when rapid cell proliferation is occurring was reviewed by DeBerardinis et al. [14]. With these mechanisms in mind, the elevated urinary levels of these metabolites during the third and fifth weeks of tumor progression appeared to reflect a tumor establishment and growth phase that resulted in a significantly changed urinary metabolome as the mice balanced maintenance of healthy cell survival in the presence of tumor establishment and growth.

## 4. Discussion and Conclusions

The goals of this study were (1) to determine if changes in urinary metabolic profiles could be used to detect the presence of a tumor growing in an orthotopic, xenograft mouse model of pancreatic cancer, and, if so, (2) to determine how early a growing tumor could be detected, i.e., if it is possible to identify early-stage biomarkers for tumorigenesis. The orthotopic, xenograft model was established by injecting human MiaPaCa-2 pancreatic cancer cells directly into the pancreata of SCID mice, which was successful in inducing tumors in 15 out of 20 mice, i.e., in 75% of the mice. Tumor formation was confirmed by palpation as well as histological data. The metabolic profiles of the orthotopic, xenograft mouse model urines collected 1 week, 3 weeks, 5 weeks, and 7 weeks post-surgery were compared to the metabolic profiles of urines collected from the control mice that had undergone sham surgery and cell growth media injected into their pancreata. Following urine collection in metabolism cages, the mice were sacrificed, serum samples were collected, and pancreata were harvested for histological analysis.

NMR-based metabolic profiling analysis of urine samples indicated that several energy metabolism pathways and pathways associated with cell proliferation and tumorigenesis were activated in the early stages of tumor initiation and growth. These pathways included glycolysis and gluconeogenesis, the TCA cycle, amino acid metabolism, pyruvate metabolism, and glyoxylate and dicarboxylate metabolism. Glycolysis and the TCA cycle metabolism supply energy intermediates that support cancer cell growth and are known to support a multitude of other pathways necessary for cancer cell survival [14,68,69]. Increased production of metabolites associated with these pathways such as citrate, acetate, alanine, and succinate were indicators of increased energy demand as a result of cancer cell proliferation, tumor establishment, and tumor growth. Increased activity in the glyoxylate and dicarboxylate metabolism pathway would also support cell proliferation, growth, and tumorigenesis, as it is involved in breaking down carbohydrates to generate other metabolites eventually involved in the biosynthesis of sugars like glucose needed to support increased cell proliferation. At the later stage of tumor development, i.e., at 7 weeks after injection of MiaPaCa-2 cells into the pancreata, the urine metabolic profiles of the control and study mice became more similar than at weeks 3 and 5 post injection, which could be attributed to the onset of tumor senescence, and these results would also be consistent with muscle wasting, albeit no data were collected to quantify muscle wasting in this study. The potential for the identified urinary metabolites to serve as early diagnostic or prognostic biomarkers for PDAC will be ultimately determined by follow-up validation studies, both using independent but similar mouse model studies, and by analyzing urine samples of human patients diagnosed with PDAC using current conventional methods.

A strength and novel aspect of this study was that the orthotopic, xenograft mouse model for pancreatic cancer progression enabled the longitudinal monitoring of tumor initiation and progression that is not possible in humans. While this study made use of a host organism that was mouse instead of human, our model does have the advantage that the tumors were composed of human pancreatic cancer cells. Because the mouse initially lacked an established tumor, this study design enabled the assessment of the earliest time at which significant metabolic profiling changes begin to occur in the urine as a consequence of tumor growth.

One limitation of this model was that the tumors did not occur spontaneously, and therefore the model lacked information about changes in urinary metabolic profiles that might accompany spontaneous tumor initiation. This model did have an advantage over orthotopic, xenograft mouse models of pancreatic cancer in which already established tumor tissue is grafted onto the mouse pancreas, since such models would not permit the monitoring of the urinary metabolic profiles prior to tumor formation or during the early stages of tumor initiation and growth. Indeed, focusing on these early time points is of utmost interest since the goal is to be able to detect pancreatic cancer at the earliest possible time points, which improves the prognosis for treatment or surgical resection. Use of the orthotopic, xenograft mouse model also had an advantage over in vitro cell line studies, which permit a careful metabolic profiling analysis of the cancer cells [70,71,72], but in vitro cell line studies alone are limited by the fact that the cancer cells are not growing in the context of the tumor environment. While the model used here had the advantage that the MiaPaCa-2 cells establish and grow in the context of a tumor microenvironment, one must keep in mind that the tumor microenvironment will be expected to be different than that in a human context, and in the presence of a fully functional immune system. In vitro cell line studies are also limited by the fact that they lack the whole organism response to tumor initiation and tumor growth that may encompass a cascade of immune responses that are entirely missing in the absence of a host organism. The fact that the research approach was that the study was conducted using only a single human pancreatic cancer cell line was another limitation of the study, whereas the universality of the conclusions would require repeating the study using other human pancreatic cancer cell lines.

A limitation of this study that was unrelated to the study design was the dependence of the metabolite assignments on databases that are known to be incomplete, which is a limitation of many NMR-based metabolic profiling studies, as we have discussed in a recent publication [37]. While the precise conclusions regarding the affected metabolic pathways are limited due to uncertainty in the resonance assignments, we have quantified the confidence in the resonance assignments using our RANCM program [33]. Despite this caveat, our conclusions that NMR-based metabolic profiling is capable of detecting early tumor progression in this orthotopic, xenograft mouse model of pancreatic cancer are strongly supported by the data presented in this study.

Characterization of this orthotopic, xenograft mouse model of pancreatic cancer represents an important step in standardizing the model for future studies that can build on these initial results. One immediate extension of this work could be to focus on complete identification of the metabolites that corresponded to resonance peaks that were determined to change significantly, but that could not be identified or confirmed with high confidence in the current study. The metabolite assignments were made based on the most up to date databases available and were based on multidimensional and heteronuclear NMR experiments, and so the assignment of the remaining significant peaks will likely require chemical structure identification for each compound, or reanalysis once more complete databases become available. Future studies could include the application of other metabolic profiling techniques to complement and compare the changes in urinary metabolism that were described here, including performing complementary liquid chromatography mass spectrometry metabolic profiling analysis in parallel with the NMR-based metabolic profiling analysis. The model could be used to generate samples for proteomics analysis of serum that was beyond the scope of this study. The model could be used to make comparisons with other mouse models of pancreatic cancer, including transgenic and xenograft mouse models of pancreatic cancer. Finally, characterization of the mouse model of pancreatic cancer used in this study is a first step toward establishing it for the future testing of new therapies for the treatment of pancreatic cancer.

Ultimately, the goal is to be able to use the knowledge gained from this study, and other similar mouse model studies, to define metabolic profiling signatures that could potentially be used for the early detection of pancreatic cancer in humans. This study illustrates that NMR-based metabolic profiling of urine has the potential to detect very early stages of PDAC initiation and progression. While this study provides strong evidence of correlations of urine metabolic profiling changes with tumorigenesis and cancer progression, further validation is needed using independent cohorts or extension to human patient diagnoses with PDAC. If validated, the approach has the added advantages that the NMR-based testing technique is non-invasive and could potentially be used for large-scale screening for early stages of disease detection. The first steps toward achieving this goal would be to use NMR-based metabolic profiling to analyze the urines of human patients when they are first diagnosed with PDAC to determine if clear urinary metabolic patterns can be identified for established disease. Eventually, the insight gained from mouse model studies such as this one should lead to hypotheses regarding potentially valuable biomarkers that will need to be validated in human clinical studies.

## Data Availability

All unnormalized and normalized binned NMR data were deposited in the public figshare database (https://figshare.com) which can be accessed at (https://figshare.com/articles/dataset/Raw_NMR_metabolic_profiling_data/28230212, accessed on 17 January 2025).

## Supplementary Materials

The following supporting information can be downloaded at: https://www.mdpi.com/article/10.3390/metabo15030142/s1, Table S1: Supplementary-Table1-1-29-25.xlsx; Table S2: compound lists-for-pathway-analysis.
