# Supplementary material for: How Early Can Pancreatic Tumors Be Detected Using NMR-Based Urine Metabolic Profiling? Identification of Early-Stage Biomarkers of Tumor Initiation and Progression in an Orthotopic Xenograft Mouse Model of Pancreatic Cancer
Source: Metabolites. 2025 Feb 20;15(3):142. doi: 10.3390/metabo15030142 (PMC11943925; doi:10.3390/metabo15030142)
Supplement: Supplementary file 1 [file metabolites-15-00142-s001.zip › compound lists-for-pathway-analysis.pdf]

Week 1 compound list

Green – significant by p-value
Blue – significant by AUC
Red – significant by VIP

1-Isovaleric acid
2- hydroxybutyrate
Ethanol
Dihydrothymine
Lactate
Alanine
Acetate
methionine sulfoxide
succinate
methylamine
2-oxoisocaproate
Dimethylamine
creatinine phosphate
creatinine
phosphorylcholine
taurine
trimethylamine N-oxide
1-methyluric acid
Ethanol
dimethylglycine
6-anhydro-beta-d-glucose
Allantoin
NADP
cis-aconitate
n-phenylacetlylglycine
glycine
Hippurate
Trigonelline
n-methylnicotinamide

Week 3 compound list

Green – significant by p-value
Blue – significant by AUC
Red – significant by VIP

1-Isovaleric acid
2- hydroxybutyrate
Ethanol
Dihydrothymine
Lactate
Alanine
Acetate
methionine sulfoxide
citrate
succinate
trimethylamine
Dimethylamine
creatinine phosphate
creatinine
choline
taurine
trimethylamine N-oxide
1-methyluric acid
glycolate
6-anhydro-beta-d-glucose
Allantoin
NADP
cis-aconitate
n-phenylacetlylglycine
glycine
Hippurate
Trigonelline
n-methylnicotinamide
dimethylglycine
taurine
imidazole

Week 5 compound list

Green – significant by p-value
Blue – significant by AUC
Red – significant by VIP

1-Isovaleric acid
2- hydroxybutyrate
Ethanol
Dihydrothymine
Lactate
Alanine
2- hydroxybutyrate
Acetate
methionine sulfoxide
citrate
succinate
trimethylamine
Dimethylamine
creatinine phosphate
creatinine
choline
phosphocholine
taurine
trimethylamine N-oxide
1-methyluric acid
6-anhydro-beta-d-glucose
NADP
cis-aconitate
n-phenylacetlyl
glycine
Hippurate
Trigonelline
n-methylnicotinamide
dimethylglycine
taurine
imidazole
benzoate

Week 7 compound list

Green – significant by p-value
Blue – significant by AUC
Red – significant by VIP

1-Isovaleric acid
2- hydroxybutyrate
Ethanol
Dihydrothymine
Lactate
Alanine
methionine sulfoxide
citrate
taurine
trimethylamine N-oxide
1-methyluric acid
6-anhydro-beta-d-glucose
NADP
cis-aconitate
n-phenylacetlylglycine
Hippurate
Trigonelline
n-methylnicotinamide
dimethylglycine
taurine
imidazole
benzoate
creatine phosphate
creatinine
